# Oral health status and practices of 6- to 7-year-old children in Amman, Jordan: a cross-sectional study

**DOI:** 10.1186/s12903-022-02342-8

**Published:** 2022-07-25

**Authors:** Ahmad Aljafari, Rawan ElKarmi, Osama Nasser, Ala’a Atef, Marie Therese Hosey

**Affiliations:** 1 Department of Paediatric Dentistry, Orthodontics, and Preventive Dentistry, School of Dentistry, The University of Jordan, Amman, Jordan; 2 Department of Dentistry, Jordan University Hospital, Amman, Jordan; 3 Centre of Oral, Clinical and Translational Science, Faculty of Dentistry, Oral and Craniofacial Sciences, King’s College London, London, UK

**Keywords:** Caries, Oral Health, Dental Health, Risk Assessment

## Abstract

**Background:**

Dental caries affects the majority of children in Jordan, with some evidence of its prevalence steadily increasing. Previous studies have shown that families struggle to establish good oral health practices. The aim of this study was to evaluate the current oral health status and practices of 6- to7-year-old children in Amman, Jordan.r

**Methods:**

A cross-sectional cohort study. The sample consisted of 6- to 7-year-old children attending six randomly selected schools in Amman, Jordan. Measures collected were: I) Caries experience (d3mft/D3MFT), II) Oral hygiene, measured using the Simplified Oral Hygiene Index, III) Dietary, toothbrushing, and dental attendance practices, measured using diaries and parental questionnaires, IV) Participants’ basic characteristics: age, education and employment. Data were analysed using SPSS20.

**Results:**

In total, 942 children were recruited. Four hundred and fifty-seven were boys, 485 were girls. Their average age was 6.5 years. Eighty-nine percent had decay in their primary teeth. Mean d3mft was 5.1(1 (range = 0–12, SD = 2.9). Only 8% of carious teeth were restored. Mean DMFT score was 0.3 (range = 0–4, SD = 0.8). Mean debris score was 1.07 (range = 0–3, SD = 0.37). Children indicated that they brush their teeth 1.6 times a day (range = 0–3, SD = 0.6). The majority (81%) were unsupervised. Sixty-seven percent of parents did not know the appropriate fluoride toothpaste concentration. Children were having 1.5 sugary snacks in-between their meals (Range = 1–6, SD = 1.1). They scored a mean of 2.5 (Range = 0–5.87, SD = 1.7) in sweetened drinks intake (recommended ≤ 1) and 2.8 (Range = 0–18.57, SD = 1.5) in non-core food intake (recommended ≤ 2) on a dietary questionnaire. Most parents (84%) indicated that their child attends the dentist only when in pain, and 18% indicated that their child is extremely afraid of dentists. Only 32% and 18% were familiar with fluoride varnish and fissure sealants, respectively. Regression analysis revealed that debris score and dental attendance were reliable predictors of caries experience.

**Conclusions:**

Six- to seven-year-old children in Amman, Jordan have a high caries experience. Most show signs of poor oral hygiene, excessive intake of cariogenic foods, and symptomatic dental attendance. Their parents lack knowledge on fluoride varnish and fissure sealants. There is a need for oral health promotion tailored to this cohort's need.

## Background

Dental caries is completely preventable, yet it is one of the most common diseases worldwide. Globally, almost 2.3 billion people suffer from caries of permanent teeth, and more than 530 million children suffer from caries of primary teeth [1]. In the Eastern Mediterranean region, the pooled prevalence of caries in the deciduous dentition in 5-year-old children is reported to be 65%, and the prevalence of the disease in the permanent dentition of 12-year-old children is 61% [2]. Early Childhood Caries (ECC) is the term used to describe caries occurring in children younger than six years of age. Worldwide data show that ECC continues to be highly prevalent, yet infrequently treated [3]. The disease negatively impacts children’s and parents’ lives and increases the cost of healthcare [4].

The aetiology of caries is multifactorial with biological, genetic, behavioural and social modifying factors [5]. Diet and feeding practices play an important role in acquisition of the infection and development of caries [6]. Significant variations in caries experience between and within countries are observed reflecting the influence of different risk factors on disease incidence [7]. ECC is higher among populations that are socially disadvantaged and particularly among children who are refugees or migrants [8]. Caregivers’ social status, poverty, ethnicity, deprivation, number of years of education, and dental insurance coverage are reported to be factors that influence the oral health practices of children and the severity of the disease [9, 10].

In Jordan, the most recent survey of school children’s oral health was in 2005. In that survey, it was found that 76.4% of 6-year-old children suffered from dental caries, with a mean dmft of 3.3 [11]. In a survey conducted in 1993, caries prevalence in 6-year-old children was reported to be 63% with a mean dmft of 2.2 [12]. It is alarming to note that caries experience in children in Jordan seems to be on the rise. In fact, current caries experience in young children might be even higher. A 2015 survey reported caries prevalence to be 77.2% in 5-year-old children with a mean dmft of 3.9 [13].

The reasons for this increase in caries prevalence and severity are not completely clear. The most recent studies on young children’s oral health practices were conducted almost twenty years ago. A study in 2005 reported that children in Jordan consume sugary foods and drinks regularly [14]. Another study reported that only half brush their teeth once or more a day, and few attend the dentist regularly [15]. Socioeconomic factors [11, 16] dietary practices [14], and dental attendance [16] were noted as factors related to caries experience. Children in public schools and those from refugee populations were particularly affected by the disease [11], and deserve special attention.

There is a need for well-designed oral health promotion activities in Jordan to reverse this worrying trend. Schools in particular can be an excellent location for delivering health promotion [17]. In light of the absence of recent data on caries prevalence and associated factors in primary school children, it is necessary to investigate those issues as part of planning any future oral health promotion. As such, the aims of this study were to establish the caries experience of 6- to 7-year-old children in public schools in Amman, Jordan, investigate the children’s dietary, oral hygiene, and dental attendance practices, and explore how those factors relate to caries experience.

## Methods

### Study design

This was a cross-sectional cohort study. The target cohort was 6- to 7-year-old children attending public schools in Amman. Jordan. The children were recruited from six randomly selected primary schools in Amman, Jordan. Data collection was performed in November and December 2019. Study design and reporting was in compliance with the ‘Strengthening the reporting of observational studies in epidemiology’ (STROBE) statement for cross-sectional studies.

### Ethical approval and consent

All research conducted in this study was in accordance with institutional and national ethical standards and the 1964 Helsinki declaration. Ethical approval was granted by Jordan University Hospital’s (JUH) Institutional Review Board (reference number: 2019/176), the Ministry of Health (reference number: 461/1122942) and the Ministry of Education (Reference number: 61298/10/3). The researchers ensured that participation was voluntary. Informed consent was obtained from parents of children taking part. Both children and parents were informed that they did not have to answer any questions and could withdraw from the study at any time without giving an explanation and without any impact on their care. Data were kept confidential and participants are not identifiable in any material published as part of the study.

### Setting and participants

There are in total 533 primary public schools in Amman, Jordan. A multi-staged cluster approach was used for sampling. First, schools that were exclusively for one gender (boys or girls), and smaller schools (with less than 120 students aged 6- to 7-years old) were excluded. One hundred and twenty-one schools remained. Next, the remaining schools were divided into two groups based on their geographical region within the city (east and west). This was to ensure schools taking part represent the two halves of the city, which are known to have different socioeconomic profiles. Finally, three schools from each geographical region were randomly selected using computer-generated numbers and invited to take part. This meant that in total six schools were included in this study. All 6- to 7-year-old children attending those schools were invited to take part. Children who declined to take part, were not in attendance on the day of the researchers’ visit, and those with learning disabilities were excluded.

### Sample size calculation

Jordan had an estimated population of 10.8 million in 2020. A total of 365,685 children were reported to be in first or second grade, out of which 219,956 (60%) attended public schools. Assuming a caries prevalence of 77% [11], and setting the confidence level at 95% and the margin of error at 3% relative to the expected proportion, a sample of 755 children was needed to ensure statistical significance.

### Measures

1. Prevalence of caries in primary and permanent teeth, recorded using dmft/DMFT scores, and percentage of caries that has been restored, calculated using the ‘care index’ (care index = f/dmf*100%).
2. Child’s oral hygiene recorded using the Simplified Oral Hygiene Index (S-OHI) [18].
3. Child’s dietary and toothbrushing practices as reported by the child using toothbrushing and diet diaries.
4. Child’s dietary practices as reported by parents using the Child Dietary Questionnaire (CDQ) [19]. The CDQ measures dietary intake in four domains: Fruits and vegetables, sweetened drinks, non-core foods, and fat from dairy.
5. Child’s toothbrushing and dental attendance practices as reported by parents using a structured questionnaire. Table 1 displays the items included in the questionnaire.

**Table 1:** Structured parental questionnaire

Demographics	Parents’ age
	Parents current employment (employed, unemployed)
	Parents’ education level (primary, secondary, college)
Oral hygiene	Brushing frequency (never, occasionally, once a day, twice or more a day)
	Age to start brushing (less than one year, 1–2 years, 2–4 years, 4–6 years, never)
	Supervision during brushing (yes, no)
	Knowledge on fluoride toothpaste concentration (yes, no)
Dental attendance	Previous dental attendance (yes, no)
	Reason for dental attendance (to treat symptoms, regular check-up)
	Age at first attendance (1 to 7)
	Perceived child dental anxiety (on a scale of 1 to 10)
	Knowledge on fluoride varnish (yes, no)
	Knowledge on fissure sealant (yes, no)

### Measure collection

The researchers visited the participating schools and distributed the consent forms to all first (6-years-old) and second grade (7-years-old) children in attendance. Next, the researchers re-visited the schools and gave all children that were willing to participate dietary diaries, toothbrushing diaries, and a parental questionnaire that records the child’s dietary practices (CDQ), oral hygiene practices, dental attendance, and parental basic characteristics. Those measures were completed at home by children and parents. Finally, the researchers would re-visit the school to collect the completed measures and perform a dental examination to record the child’s caries experience (dmft/DMFT) and S-OHI scores.

### The dental examination

Researchers (AA) and (RE), who are Paediatric Dentistry Consultants, performed the dental examination. The examination took place in a well-lit room in the schools and the recommendations outlined by the WHO’s manual were followed [20]. The examiners noted the child’s decayed, missing due to caries, and filled teeth in both primary (dmft) and permanent dentition (DMFT). Caries was recorded at the ‘obvious decay experience’ level, defined as ‘caries that can be visualised through the enamel or lesions where it has advanced to form a frank cavity’ [21]. The examiners also recorded the children’s oral hygiene according to the S-OHI [18]. The S-OHI includes recording debris and calculus accumulation on up to six tooth surfaces with each surface given a score from zero to three for debris and for calculus. To ensure examiner calibration, examiners first discussed and agreed upon their approach and criteria for scoring. Then they examined six children together in a set-up similar to that of the study. Each examiner recorded dmft/DMFT and S-OHI scores for the children, then they compared their scores, discussed, and re-examined any differences until a consensus-was reached. To establish inter-rater reliability, each examiner separately recorded the presence of caries and S-OHI scores for another sample of six children. The scores were entered into SPSS 20 and Cohen’s Kappa tests were performed. Kappa scores were 0.88 for caries and 0.70 for S-OHI score, which were deemed acceptable. To ensure intra-rater reliability, the children were re-examined after one week. Kappa score for caries for both examiners was 0.94.

### Pilot study

A pilot study was performed prior to the commencement of the main study to ensure the readability and relevance of the child diaries and parental questionnaires. The pilot study had a qualitative design. Seventeen pairs of 6- to 7-year-old children and parents attending the Paediatric Dental clinic at JUH’s Dental department took part. They were asked to read the questionnaires and provide feedback. Researcher observations, child feedback, and parental feedback were recorded then categorised accordingly. Data were then analysed using a simple content analysis. The results showed that the readability and relevance of the questionnaires was satisfactory.

### Statistical analysis

All data were entered into SPSS 20. Descriptive statistics (mean, standard deviation) were recorded for continuous variables (dmft/DMFT, S-OHI, CDQ, Brushing and sugar intake frequency, dental anxiety, and parents’ age). Frequencies were recorded for categorical variables (parental education and employment, oral hygiene habits, dental attendance habits, and parental knowledge on preventive treatments). dmft scores were dichotomised to presence (dmft > 0) and absence (dmft = 0) and multiple binary logistic regression was used to investigate predictors for caries experience in primary teeth. Odds ratios for predictors were calculated and presented with 95% confidence intervals. Only independent variables found to be statistically significant (*P* ≤ 0.05) on a bivariate binary logistic regression level were included in the multiple regression model for analysis. Forward stepwise multiple linear regression was used to investigate predictors for dmft score. Beta coefficients and 95% confidence intervals for each predictor and R-squared value for the model were calculated and presented. Only independent variables that were found to be statistically significant (*P* ≤ 0.05) on a bivariate linear regression level were included in the multiple regression model for analysis. Categorical variables that included more than two categories (parental education, toothbrushing frequency, age to start toothbrushing, age at first dental attendance) were dichotomised prior to regression analysis. A one-way ANOVA test was used to explore whether parents’ and children’s reports on diet were correlated and a Pearson’s correlation test was used to evaluate whether children’s and parents’ reports on toothbrushing were consistent. The significance level for all tests was set at *P* ≤ 0.05.

## Results

### Recruitment

Data collection took place in November and December 2019. Children in six schools took part. A total of 1009 children were determined to fit the inclusion criteria, of which 942(93%) took part. The remaining 67 children (7%) were excluded due to being absent on the day of the researchers’ visit or not providing consent.

Eight hundred and thirty two children (89%) completed a dental examination and had their dmft/DMFT and S-OHI score recorded. Parents of 680 children (72%) returned completed parental questionnaires. Six hundred and four children (64%) returned completed diet diaries, and 567 children (60%) returned completed toothbrushing diaries.

### Sample description

The children’s age and gender, and their parents’ age, education, and employment status are summarised in Table 2.

**Table 2:** The participants

Child	
Age	Mean = 6.5 (SD = 0.5; range = 6-7)
School class	
First grade	439 (47%)
Second grade	503 (53%)
Gender	
Male	456 (49%)
Female	485 (51%)

Mother	
Age	Mean = 34.5 (SD = 6.1; range = 20-58)
Employment	
Unemployed	500 (75%)
Employed	167 (25%)
Education	
Primary	167 (25%)
Secondary	266 (40%)
University	236 (35%)

Father	
Age	Mean = 40.9 (SD = 7.7; range = 20-90)
Employment	
Unemployed	515 (78%)
Employed	144 (22%)
Education	
Primary	194 (29%)
Secondary	268 (41%)
University	197 (30%)

### Dental caries

Seven hundred and forty-four of those examined (89%) had caries in at least one primary tooth. The mean dmft score was 5.1 (range 0–12, SD = 2.9). The mean number of decayed primary teeth was 4.4 (range 0–12, SD 2.9), while the mean number of primary teeth prematurely lost was 0.3 (range 0–3, SD = 0.6) and the mean number of those filled was 0.4 (range 0–7, SD = 1.1). Care index (f/dmft*100%) for the sample was very low (8%).

Seven hundred children had at least one permanent first molar erupted (84%). One hundred and eight of them (15%) had decay in at least one permanent molar. The mean DMFT score for the sample was 0.3 (Range 0–4, SD = 0.8). The mean number of decayed permanent teeth was 0.3 (range 0–4, SD = 0.8), while none were deemed to be extracted and the mean number of those restored was negligible (0.01).

### S-OHI scores

The average debris score for the children examined was 1.07 (Range 0–3, SD = 0.37). None of the children had calculus at the time of examination. Table 3 summarises the children’s caries experience and debris scores according to their demographic characteristics.

**Table 3:** Caries experience and debris scores according to demographic characteristics

	dmft	DMFT	debris score
*Child*			
School class			
First grade	4.77 (SD = 2.92)	0.12 (SD = 0.48)	1.00 (SD = 0.37)
Second grade	5.32 (SD = 2.92)	0.44 (SD = 0.92)	1.13 (SD = 0.36)
Gender			
Male	5.15 (SD = 2.91)	0.23 (SD = 0.69)	1.08 (SD = 0.37)
Female	4.99 (SD = 2.96)	0.36 (SD = 0.85)	1.06 (SD = 0.37)
*Mother*			
Employment			
Unemployed	4.76 (SD = 2.99)	0.25 (SD = 0.71)	1.04 (SD = 0.36)
Employed	5.47 (SD = 2.62)	0.33 (SD = 0.84)	1.10 (SD = 0.38)
Education			
Primary	5.16 (SD = 2.87)	0.34 (SD = 0.81)	1.09 (SD = 0.33)
Secondary	4.79 (SD = 3.09)	0.30 (SD = 0.82)	1.09 (SD = 0.36)
University	4.98 (SD = 2.75)	0.21 (SD = 0.64)	1.02 (SD = 0.40)
*Father*			
Employment			
Unemployed	5.22 (SD = 2.72)	0.26 (SD = 0.73)	1.09 (SD = 0.37)
Employed	4.87 (SD = 2.96)	0.27 (SD = .075)	1.05 (SD = 0.36)
Education			
Primary	4.83 (SD = 2.85)	0.35 (SD = 0.87)	1.13 (SD = 0.37)
Secondary	5.04 (SD = 3.01)	0.29 (SD = 0.77)	1.05 (SD = 0.33)
University	4.03 (SD = 2.90)	0.21 (SD = 0.62)	1.03 (SD = 0.40)

### Oral hygiene practices

Five hundred and sixty-seven children (60%) completed toothbrushing diaries. Four hundred and ninety-nine children (88%) indicated that they brush at least once a day. The children’s mean toothbrushing frequency was 1.6 times a day (range 0–3, SD = 0.60). Six hundred and seventy-nine parents (72%) completed a questionnaire on the child’s oral hygiene practices. When asked about the child’s frequency of brushing, 243 parents (36%) indicated that their child brushes at least twice a day, while 315 (47%) said that the child brushes at least once a day. Seventy-seven (11%) noted that their child brushes only occasionally and 44 (6%) said their child never brushes. Using a one-way ANOVA test, it was noted that toothbrushing frequency reported by the child and by the parent were significantly correlated (*P* < 0.001*).

When asked about the age at which their child started brushing their teeth, only ten parents (1%) indicated that the child started before the age of one year. Ninety-six (14%) said that they started between the age of one and two, 215 (32%) that they started between the ages of two and four, 306 (45%) that they started between the ages of four and six, and 52 (8%) that their child still does not brush his/her teeth. Five hundred and thirty-five parents (81%) said that their child brushes unsupervised. Four hundred and forty-four parents (67%) said that they do not know the appropriate fluoride toothpaste concentration to use in their children.

### Dietary practices

Six hundred and four children (64%) completed three-day dietary diaries. Those diaries were analysed to determine the frequency of sugar intake in-between meals. Children on average had 1.5 sugary snacks a day (Range: 1–6, SD = 1.1). Six hundred and eighty parents (72%) completed the CDQ. The average score for fruit and vegetables intake (Recommended is ≥ 14) was 13.5 (Range 0–27.9, SD = 5.5). The average score for fat from dairy (Recommended is 0) was seven (Range 0–20, SD = 4.9). The average score for sweetened drinks (recommended is ≤ 1) was 2.5 (Range 0–5.87, SD = 1.7). Finally, the average score for non-core foods (recommended is ≤ 2) was 2.8 (Range 0–18.57, SD = 1.5). There was weak but significant correlation between the number of sugary snacks that a child reported on their diary and the non-core foods and sweetened drinks scores that their parent reported (Pearson Correlation = 0.24; *P* < 0.001*).

### Dental attendance

Parents of 678 children (72%) provided information on their child’s dental attendance. Four hundred and sixty-seven parents (69%) said that their child has been to the dentist before, while 211(31%) indicated that the child never visited a dentist. Those that attended were on average 4.8 years old (Range 1–8, SD = 1.3) on their first visit. The majority (84%) said that their child attended the dentist for treatment of symptoms. Only 75 children (16%) were said to have attended for a regular check-up. Four hundred and fifty six parents (68%) were not familiar with fluoride varnish application, while 217 (32%) were. Meanwhile, 549 parents (82%) were not familiar with fissure sealant application, while 123 (18%) were.

### Dental anxiety

Parents of 645 children (69%) provided data on their child’s anxiety when attending the dentist. Asked to indicate on a scale of one to ten how anxious their child is when attending the dentist (with one being not anxious at all and ten being extremely anxious), the mean score given by the parents was 4.9 (Range 1–10, SD = 3.2). Score ‘1’ was the most frequently chosen score and was selected by 27% of the parents, while score ‘5’ was selected by 26% and score’10’ by 18%.

### Predictors of caries experience in primary teeth

Table 4 displays oral hygiene, dietary and dental attendance practices according to caries presence/absence. When bivariate binary logistic regression was performed, debris score, dental fear, and pattern of dental attendance were found to be statically significant predictors of caries presence in primary teeth. After a stepwise multiple logistic regression, only debris score and pattern of dental attendance remained as statistically significant. When bivariate linear analyses were performed, the following were found to be statistically significant predictors of severity of caries experience in primary teeth (dmft score): debris score, child age, mothers employment, history of visiting the dentist, pattern of dental attendance and dental fear. Those variables were analysed using multiple linear regression. Debris score, history of visiting the dentist, and reason for dental attendance were the only variables that remained statistically significant. The results of the regression analysis are summarised in Table 5.

**Table 4:** Oral hygiene, dietary and dental attendance practices according to caries presence/absence and overall

	Caries present	Caries absent
*Oral hygiene practices*		
Mean debris score	1.10 (SD = 0.36)	0.89 (SD = 039)
Mean toothbrushing frequency	1.6 (SD = 0.6)	1.5 (SD = 0.6)
Brushes at least twice daily (%)	37%	32%
Brushes supervised (%)	19%	26%
Parent unsure of recommended fluoride toothpaste concentration (%)	66%	63%
Started brushing before the age of two (%)	15%	19%
*Dietary practices*		
Mean number of in-between meals sugary snacks	1.5 (SD = 1.1)	1.4 (SD = 0.9)
Mean fruit and vegetables score (Recommended is ≥ 14)	13.5 (SD = 5.5)	13.2 (SD = 5.7)
Mean sweetened drinks score (recommended is ≤ 1)	2.4 (SD = 1.7)	2.6 (SD = 1.7)
Mean fat from dairy score (Recommended is 0)	7.0 (SD = 4.9)	7.0 (SD = 5.0)
Mean non-core foods score (recommended is ≤ 2)	2.8 (SD = 1.6)	2.6 (SD = 1.4)
*Dental attendance*		
Mean dental anxiety score (1–10)	5.0 (SD = 3.2)	4.1 (SD = 3.1)
Mean age at first attendance	4.8 (SD = 1.3)	4.7 (SD = 1.5)
Attended the dentist before (%)	71%	63%
Attends the dentist regularly (%)	15%	33%
Parent not familiar with fluoride varnish (%)	68%	62%
Parent not familiar with fissure sealant (%)	81%	82%

**Table 5:** Predictors of caries experience in primary teeth

Independent variable	OR	95% CI	*P*-value
Caries presence			
Debris score	3.74	1.41–9.94	*P* = 0.008
Dental attendance			
Regular (reference)			
Symptomatic	2.47	1.20–5.07	*P* = 0.014

Independent variable	BETA	SE	95% CI	*P*-value
Caries severity				
Debris score	2.24	0.35	1.54–2.93	*P* < 0.001
Dental visits				
No previous visit (reference)				
Visited dentist before	2.32	0.70	0.94–3.70	*P* = 0.001
Dental attendance				
Regular (reference)				
For symptoms	1.35	0.36	0.63–2.06	*P* < 0.001
Intercept	− 0.79	0.83	− 2.42–0.85	*P* = 0.345
Adjusted R^2 ^= 0.16, F = 14.44, *P *< 0.001*				

## Discussion

The findings of this study demonstrate that the prevalence of dental caries in school children in Amman, Jordan is very high, and confirms the worrying trend of increasing caries prevalence in children in Jordan over the past 30 years [11–13]. Moreover, the vast majority of caries was left untreated. This can significantly impact the child's and family's quality of life [22]. Dental caries is completely preventable, and significant reductions in prevalence of childhood dental caries have been achieved in other high-caries-risk cohorts through tailored oral health interventions and policies [23]. As such, there is an urgent need for oral health promotion in Jordan. Children and families taking part in this study reported poor dietary practices, a deficient approach to toothbrushing, and a lack of regular access to dental care and preventive treatments. These are all issues that will need to be accounted for and addressed in future oral health promotion planning.

Children in this study reported toothbrushing frequency that is lower than the recommended twice a day. In addition, they were mostly unsupervised during toothbrushing by their parents. Supervision of brushing in this age group is important and recommended in an evidence-based guideline [24]. Suboptimal brushing frequency and lack of supervision might explain the fact that most children in this study had debris on their teeth at the time of examination. It is also possible that some participants were overestimating their brushing frequency to provide what they think is a socially desirable answer. Future oral health promotion efforts need to ensure that both parents and children are targeted and motivated to brush twice daily under supervision. A good way to ensure that children brush their teeth supervised at least once daily would be to introduce a national toothbrushing programme at schools. Those programmes have proven successful in other countries and support families that have difficult socioeconomic circumstances [25].

Parents were mostly unsure of the correct fluoride toothpaste concentration. Only a few indicated that they would use adult concentration toothpaste (1350–1500 ppm), despite it being the recommended dosage for high-caries-risk children [24]. There is a consensus that the minimum concentration of fluoride in children's toothpaste should be 1000 ppm [26]. There is a need to include guidance on fluoride concentration in public oral health education messages, and to ensure that children’s toothpastes available in the market comply with the minimum concentration. Manufacturers also need to prominently display this information on their products to make the selection process easier for the parents.

Children in this study reported an average of one and a half sugar-containing snacks a day. Furthermore, they were consuming sweetened drinks and non-core foods in amounts exceeding recommendations. This is in line with the results of previous studies in Jordanian children [14, 27]. Sugar consumption is strongly linked to caries [28], and frequency of intake is of particular importance [29]. As such, it is important to promote healthy eating in those families. One on one dietary advice remains the gold standard [30], but other approaches, such as the use of social media, mobile applications, and video games [31] can be explored. It is important that this is also accompanied with policies to restrict the availability of cariogenic foods in schools and facilitate children’s access to healthy alternatives.

The majority of participants indicated that they attend the dentist only when in pain. This confirms the results of previous studies in Jordan [15]. In addition, most cavities were not treated at the time of examination. Moreover, about one fifth of children in this study were deemed extremely fearful of dental treatment. This fact, combined with the high number of decayed teeth noted in this study, suggest that many of these children would end up needing treatment by a Paediatric specialist under General Anaesthesia. This is a common but relatively serious procedure for a disease that could ideally be completely prevented. About 41% of Jordanians are covered with public health insurance [32] that covers dental care in children. Moreover, all children aged six or younger can receive dental care for free at public centres and hospitals. Dentists in Jordan previously indicated that they feel that parental motivation is the most important factor in providing dental care for children [33]. It is important that future oral health promotion encourages regular attendance every three to six months, as recommended for children in this age group, and that we continue to explore barriers to access of dental care and how those can be addressed.

Most parents were unfamiliar with fluoride varnish and fissure sealants. Those preventive treatments are recommended by various organisations and have proven their effectiveness in caries prevention [34, 35]. In a previous study, dentists in Jordan indicated that these treatments are available but highlighted parental motivation as a crucial factor in patient selection [33]. Raising public awareness about the benefits of these treatments is necessary. Fluoride varnish in particular is very easy to apply and can be done outside of the dental setting [36]. Hence, it can be used as part of wider school oral health promotion programmes.

When our regression models were finalised, only the level of debris at the time of examination and dental attendance patterns remained as predictors of dental caries presence and severity. Child- and parent-reported oral hygiene practices were not a significant predictor. This might be due to the fact that the presence of debris is an objective indicator of unsatisfactory oral hygiene practices, while participant-completed measures can be affected by what's perceived as a socially desirable answer. The relationship between caries and dental attendance patterns is understandable [15]. Children who attended the dentist regularly were less likely to have caries than those who did not. This can be both a cause and an effect; undergoing regular dental care can prevent dental caries, and the absence of dental caries in turn reduces the chances of needing to visit the dentist to relieve pain. None of the dietary variables were good predictors of caries presence or severity. It is possible that this was due to dietary measures being self-reported. It is also possible that this is because those measures report on sugar intake frequency and amount, but do not factor in other practices related to diet, such as time between food consumption and brushing, type of food (sticky, non-sticky), and consumption of protective foods. Dietary factors have been noted as less impactful since the mass introduction of fluoride [37], but remain a very important factor in oral health and caries prevention. Parents’ education and employment were also not a significant predictor of caries. This is most likely due to the fact that our sample consisted of children in public schools in an urban area, in a developing country. As such, most parents in the sample, even those educated and employed, can be assumed to have relatively difficult socioeconomic circumstances in comparison with those that are more affluent in developing and developed countries. In fact, the attendance of a public school in Jordan was itself considered a predictor of poor socioeconomic status in a previous study [11].

This study had its limitations. First, dietary, oral hygiene, and dental attendance measures were self-reported and as such carry the risk of being influenced by what the participants thought were socially desired answers. However, child diaries have been found reliable before [38], and in this study it was encouraging to note that parents’ and children’s reports on toothbrushing and sugar consumption were significantly correlated, suggesting that these measures can be somewhat reliable and consistent. Second, the questionnaires used were not validated in this population. However, they have been validated for use in children of the same age in different populations [19] and were piloted prior to their use in the study. Third, the sample was drawn from a relatively small number of schools and did not include private schools. However, care was taken during sampling so that the participating schools are chosen randomly from areas with differing socioeconomic status. Finally, the examination was performed outside of the dental setting, meaning that diagnostic aids, such as radiography, were not used. However, the WHO guidelines [20] for examination in a non-clinical setting were followed to ensure quality.

The study also had its strengths. First, it provided the first update on this populations' oral health status, oral hygiene and dietary practices, and dental attendance in almost two decades. In addition, it identified particular deficiencies in this population’s oral health knowledge and practices, and addressing those can be incorporated into future efforts for oral health promotion. The sample recruited seems to be representative of the Jordanian population in terms of education and employment [39], and the results can be generalised in the Jordanian child population. Studies from other countries in the region report similar trends in dental caries and the findings of this study might apply to similar populations in the Eastern Mediterranean and North Africa regions [40].

## Conclusions

In conclusion, children in primary public schools in Amman, Jordan are severely affected by dental caries. The majority have debris on their teeth and have multiple cavities that are untreated. Their families report deficiencies in their dietary and oral hygiene practices. They access dental care only to relieve pain and are not familiar with preventive treatments, such as fluoride varnish and fissure sealant. Future oral health promotion initiatives need to focus on this population. Programmes inside and outside schools need to promote a healthy diet, appropriate toothbrushing, and regular dental attendance. In addition, studies to explore barriers to dental care access and how those can be resolved are needed.

## Data Availability

The data that support the findings of this study are available from the corresponding author (AA) upon reasonable request.

## Abbreviations

CDQ: Child’s dietary questionnaire
Dmft: Decayed, missing, and filled primary teeth
DMFT: Decayed, missing, and filled permanent teeth
ECC: Early childhood caries
JUH: Jordan university hospital
S-OHI: Simplified oral hygiene index
STROBE: Strengthening the reporting of observational studies in epidemiology

## References

[1] James SL, Abate D, Abate KH, Abay SM, Abbafati C, Abbasi N, Abbastabar H, Abd-Allah F, Abdela J, Abdelalim A, Abdollahpour I (2018). Global, regional, and national incidence, prevalence, and years lived with disability for 354 diseases and injuries for 195 countries and territories, 1990–2017: a systematic analysis for the global burden of disease study 2017. Lancet.

[2] Kale S, Kakodkar P, Shetiya S, Abdulkader R (2020). Prevalence of dental caries among children aged 5–15 years from 9 countries in the Eastern Mediterranean region: a meta-analysis. East Mediterr Health J.

[3] Tinanoff N, Baez RJ, Diaz Guillory C, Donly KJ, Feldens CA, McGrath C, Phantumvanit P, Pitts NB, Seow WK, Sharkov N, Songpaisan Y (2019). Early childhood caries epidemiology, aetiology, risk assessment, societal burden, management, education, and policy: Global perspective. Int J Pediatr Dent.

[4] Casamassimo PS, Thikkurissy S, Edelstein BL, Maiorini E (2009). Beyond the dmft: the human and economic cost of early childhood caries. J Am Dent Assoc.

[5] Seow WK (1998). Biological mechanisms of early childhood caries. Community Dent Oral Epidemiol.

[6] Palmer CA, Kent R, Loo CY, Hughes CV, Stutius E, Pradhan N, Dahlan M, Kanasi E, Arevalo Vasquez SS, Tanner AC (2010). Diet and caries-associated bacteria in severe early childhood caries. J Dent Res.

[7] Do LG (2012). Distribution of caries in children: variations between and within populations. J Dent Res.

[8] Edelstein BL (2006). The dental caries pandemic and disparities problem. BMC Oral Health.

[9] Aida J, Ando Y, Aoyama H, Tango T, Morita M (2006). An ecological study on the association of public dental health activities and sociodemographic characteristics with caries prevalence in Japanese 3-year-old children. Caries Res.

[10] Ramos-Gomez FJ, Weintraub JA, Gansky SA, Hoover CI, Featherstone JD (2002). Bacterial, behavioral and environmental factors associated with early childhood caries. J Clin Pediatr Dent.

[11] Rajab LD, Petersen PE, Baqain Z, Bakaeen G (2014). Oral health status among 6-and 12-year-old Jordanian schoolchildren. Oral Health Prev Dent.

[12] Hamdan MA, Rock WP (1993). Dental caries experience in Jordanian and English schoolchildren. Community Dent Health.

[13] Rajab LD, Abdullah RB (2020). Impact of dental caries on the quality of life of preschool children and families in Amman Jordan. Oral Health Prev Dent.

[14] Sayegh A, Dini EL, Holt RD, Bedi R (2005). Oral health, sociodemographic factors, dietary and oral hygiene practices in Jordanian children. J Dent.

[15] Rajab LD, Petersen PE, Bakaeen G, Hamdan MA (2002). Oral health behaviour of schoolchildren and parents in Jordan. Int J Paediatr Dent.

[16] Rajab LD, Hamdan MA (2002). Early childhood caries and risk factors in Jordan. Community Dent Health.

[17] Kwan SY, Petersen PE, Pine CM, Borutta A (2005). Health-promoting schools: an opportunity for oral health promotion. Bull World Health Organ.

[18] Greene JC, Vermillion JR (1964). The simplified oral hygiene index. J Am Dent Assoc.

[19] Magarey A, Golley R, Spurrier N, Goodwin E, Ong F (2009). Reliability and validity of the children's dietary questionnaire; a new tool to measure children's dietary patterns. Int J Pediatr Obes.

[20] World Health Organization (2013). Oral health surveys basic methods.

[21] Pitts N, Chadwick B, Anderson T. Children’s Dental Health Survey 2013. Report 2: dental disease and damage in children: England, wales and Northern Ireland. Health and social care information centre; (2015). https://digital.nhs.uk/data-and-information/publications/statistical/children-s-dental-health-survey/child-dental-health-survey-2013-england-wales-and-northern-ireland. Accessed 1 May 2022

[22] Abanto J, Tsakos G, Paiva SM, Carvalho TS, Raggio DP, Bönecker M (2014). Impact of dental caries and trauma on quality of life among 5- to 6-year-old children: perceptions of parents and children. Community Dent Oral Epidemiol.

[23] Fraihat N, Madae'en S, Bencze Z, Herczeg A, Varga O (2019). Clinical effectiveness and cost-effectiveness of oral-health promotion in dental caries prevention among children: systematic review and meta-analysis. Int J Environ Res Public Health.

[24] Public Health England. Delivering Better Oral Health: An evidence-based toolkit for prevention, Third Edition. London; 2017.

[25] Macpherson LM, Anopa Y, Conway DI, McMahon AD (2013). National supervised toothbrushing program and dental decay in Scotland. J Dent Res.

[26] Walsh T, Worthington HV, Glenny AM, Marinho VC, Jeroncic A (2019). Fluoride toothpastes of different concentrations for preventing dental caries. Cochrane Database Syst Rev.

[27] ElKarmi R, Aljafari A, Eldali H, Hosey MT (2019). Do expectant mothers know how early childhood caries can be prevented? a cross-sectional study. Eur Arch Paediatr Dent.

[28] Moynihan PJ, Kelly SA (2014). Effect on caries of restricting sugars intake: systematic review to inform WHO guidelines. J Dent Res.

[29] Duggal MS, Toumba KJ, Amaechi BT, Kowash MB, Higham SM (2001). Enamel demineralization in situ with various frequencies of carbohydrate consumption with and without fluoride toothpaste. J Dent Res.

[30] Harris R, Gamboa A, Dailey Y, Ashcroft A (2012). One-to-one dietary interventions undertaken in a dental setting to change dietary behaviour. Cochrane Database Syst Rev.

[31] Aljafari A, Gallagher JE, Hosey MT (2017). Can oral health education be delivered to high-caries-risk children and their parents using a computer game?–A randomised controlled trial. Int J Paediatr Dent.

[32] Ravishankar N, Gausman J. Analysing equity in health utilization and expenditure in Jordan-with focus on maternal and child health services. ThinkWell/UNICEF, 2016. https://thinkwell.global/wp-content/uploads/2016/10/Thinkwell-Jordan-Report-FINAL_August31.pdf. Accessed 1 May 2022.

[33] Aljafari A, ElKarmi R, Kussad J, Hosey MT (2021). General dental practitioners' approach to caries prevention in high-caries-risk children. Eur Arch Paediatr Dent.

[34] Ahovuo-Saloranta A, Forss H, Walsh T, Hiiri A, Nordblad A, Mäkelä M, Worthington HV (2013). Sealants for preventing dental decay in the permanent teeth. Cochrane Database Syst Rev.

[35] Marinho VC, Worthington HV, Walsh T, Clarkson JE (2013). Fluoride varnishes for preventing dental caries in children and adolescents. Cochrane Database Syst Rev.

[36] Turner S, Brewster L, Kidd J, Gnich W, Ball GE, Milburn K, Pitts NB, Goold S, Conway DI, Macpherson LM (2010). Childsmile: the national child oral health improvement programme in Scotland. Part 2: monitoring and delivery. Br Dent J.

[37] Touger-Decker R, van Loveren C (2003). Sugars and dental caries. Am J Clin Nutr.

[38] Gil GS, Morikava FS, Santin GC, Pintarelli TP, Fraiz FC, Ferreira FM (2015). Reliability of self-reported toothbrushing frequency as an indicator for the assessment of oral hygiene in epidemiological research on caries in adolescents: a cross-sectional study. BMC Med Res Methodol.

[39] Department of Statistics (Jordan) and ICF International. Jordan population and family health survey 2012. Calverton, Maryland, USA: Department of Statistics and ICF International; 2013. https://dhsprogram.com/pubs/pdf/fr282/fr282.pdf. Accessed 1 May 2022.

[40] Elamin A, Garemo M, Mulder A (2021). Determinants of dental caries in children in the Middle East and North Africa region: a systematic review based on literature published from 2000 to 2019. BMC Oral Health.

